# The Effect of Compost-Bedded Pack Barns on Claw Health and Lameness in Dairy Herds in Southern Germany

**DOI:** 10.3390/ani15091347

**Published:** 2025-05-07

**Authors:** Phillip Andreas Guhl, Adrian Steiner, Lisa Bachmann, Maike Heppelmann

**Affiliations:** 1 Clinic for Cattle, University of Veterinary Medicine Hannover, Foundation, 30173 Hannover, Germany; 2 Clinic for Ruminants, Vetsuisse Faculty, University of Bern, 3012 Bern, Switzerland; adrian.steiner@unibe.ch; 3 Faculty of Agriculture and Food Sciences, University of Applied Science Neubrandenburg, 17033 Neubrandenburg, Germany; bachmann@hs-nb.de

**Keywords:** compost-bedded pack barn, lameness, claw health, dairy cow

## Abstract

The aim of this study was to determine claw health and lameness prevalence in cows housed in CBPs in southern Germany. Eight farms that housed their dairy cows in CBPs were visited for data collection once in the warm season and once in the cold season between January and December 2023. The first visit was during hoof trimming of the herd to identify claw disorders, score lameness, and assess the bedded pack resting area. Lameness was scored again and the bedded pack resting area assessed at the second visit. To compare claw health at cow and farm levels, a cow claw score (CCS) and a farm claw score (FCS) were calculated using geometric severity scores. The prevalence of lameness at cow level was 9.4% in the cold season and 11.1% in the warm season, which were lower than values reported in studies that investigated cubicle free-stall barns. The low prevalences of lameness and claw disorders were reflected in a CCS of 8 and FCS of 9, which are defined as excellent. Based on our results, CBPs are associated with low lameness prevalence and favourable claw health.

## Simple Summary

The recognition of CBPs as a beneficial housing system continues to grow, particularly regarding cow health and welfare. Previous studies have shown a lower prevalence of lameness and good claw health for cows housed in CBPs. However, further research is needed to explore its impacts more comprehensively, making this study relevant. Therefore, this study determined the claw health and prevalence of lameness in cows housed in eight CBPs in Germany. The prevalence of lameness in the study was 9.4% in the cold and 11.1% in the warm season, which were considerably lower than results from previous studies in conventional free-stall barns. In addition, the claw health of the scored cows was excellent, which was reflected in a low claw health score at cow and farm levels.

## 1. Introduction

Lameness is one of the most common production diseases in dairy cows [1,2,3], impacting animal welfare and the economic performance of farms. The latter includes losses attributable to reduced milk production, poor reproductive performance, and high culling rates [4,5,6]. Welfare concerns encompass pain and stress [7] as well as changes in the eating, lying, and activity behaviours in lame cows [8].

Based on the results of 53 studies from around the world, the prevalence of lameness in dairy herds ranges from 5.1% to 45% with a mean of 22% [9]. The PraeRi study analysed animal health in 765 dairy farms in southern (260 farms), northern (253 farms), and eastern (252 farms) Germany from 2016 to 2020. Data on housing, hygiene, feeding, animal health, and management were collected. The recorded prevalence of lameness in cubicle barns was 23.6% in southern, 24.6% in northern, and 41.1% in eastern Germany [10]. More than 90% of lameness cases were attributable to claw disorders [11]. A recent study evaluated the claw health of cattle from 508 Austrian farms using data sets collected by hoof trimmers. White line disease (WLD) and heel horn erosion (HHE) were the most common foot and claw disorders. In herds with endemic digital dermatitis (DD), DD was the third most common claw disease [12].

Wet concrete surfaces and uncomfortable lying surfaces have a negative impact on lameness in cows housed in cubicle barns [13,14]. Other factors associated with lameness are related to the individual cow, nutrition, cow comfort, barn design, and herd management [13,14].

In recent years, alternative housing systems such as compost-bedded pack barns (CBPs) have become increasingly popular. A CBP is a loose housing system with a concrete walkway and a bedded pack resting area consisting of sawdust and fine dry wood shavings. The bedding material is tilled twice daily to promote biodegradation of manure, urine, and wood shavings under aerobic conditions. Tilling also provides a fresh surface and aids in drying of the bedding [15].

Compared with cubicle housing, CBPs have been associated with increased milk production, decreased somatic cell counts (SCCs), and reduced mastitis rates [16,17]. The reproductive performance of cows that were moved into CBPs also improved with decreased calving intervals and increased heat detection and pregnancy rates [16,17]. Improvements in animal welfare involve many factors, culminating in reduced culling rates and increased longevity [16,18]. Compost-bedded pack barns promote natural behaviour with higher lying times per day compared with free-stall barns [19,20,21]. Several studies have shown that cows housed in CBPs have a lower prevalence of hock lesions and lameness compared with cows housed in other types of free-stall barns [16,22,23]. In Austria, CBPs were associated with better claw health as evidenced by a lower prevalence of claw lesions, including white line disease, heel horn erosion, concave dorsal wall, and interdigital hyperplasia [24].

The aim of this study was to evaluate the prevalence of lameness and claw health in dairy cows housed in CBPs in Germany. Based on the results of previous studies, we hypothesise that the prevalences of lameness and claw disorders will be lower in CBPs than in conventional housing systems.

## 2. Materials and Methods

### 2.1. Selection of Farms

Large breeding organisations, cattle auction barns, barn construction companies, and hoof trimmers were contacted to help locate CBPs in Germany. Inclusion of farms in the study was based on the following prerequisites: All lactating cows were housed in CBPs, CBPs had been used as the housing system for a minimum of two years, cows had no access to pasture, and hoof trimming was carried out at least once a year for the entire dairy herd. Of 30 CBPs identified, only eight farms fulfilled the requirements; seven CBPs were in Bavaria, and one was in Baden-Württemberg.

The main business focus of the eight farms was dairy farming; one farmer had an organic dairy farm, and two farmers sold their products directly to market.

The farms housed 64.5 ± 8.5 (median ± MAD) lactating dairy cows, 10.8 ± 4.6 (mean ± SD) dry cows, and 87.8 ± 38.6 (mean ± SD) young stock. The number of cows increased in 2022 by more than 10% on three farms and remained constant on the other five farms. The dominant breed was German Fleckvieh (82.2%), followed by Holstein Friesian (13.4%), and other breeds or crossbred cows (4.4%). All farmers used an automatic milking system (AMS). Milk production was 8417.4 ± 879.7 kg (mean ± SD), with 4.19 ± 0.14% (mean ± SD) fat, 3.58 ± 0.08% (median ± MAD) protein, and an SCC of 243,125 ± 109,919 cells/mL (mean ± SD). Lactating cows were in lactation number 2 ± 1 (median ± MAD). All cows were fed a total mixed ration (TMR) using a TMR mixer on seven farms and an automatic feeding system on one farm.

### 2.2. Farm Visits

Each farm was visited twice between January 2023 and December 2023 by two observers, one of whom attended both visits. Each of the two visits comprised two consecutive days. The first visit took place at the same time as hoof trimming, while the second occurred the following season. The year was divided into two seasons: the cold (December to April) and the warm (June to September) seasons. When the first visit occurred during the cold season, the second was in the warm season and vice versa. On the first day of the first visit, the observers completed a questionnaire covering the topics, such as general farm data, compost management, claw health, and farmers’ opinion on certain aspects, and assessed barn construction characteristics, housing conditions, and compost quality. Claw lesions were documented during claw trimming on the second day. Lameness was scored on the first day or before claw trimming. Lameness scoring and the assessment of barn construction, housing conditions, and compost quality were repeated on the second visit. In the cold season, 593 cows and in the warm season, 613 cows were scored during the farm visits.

In consultation with the Animal Protection Committee of the University of Veterinary Medicine Hannover (TiHo), ethical review and approval were waived for this study as the methods of the study are not considered animal experiments.

Herd data from HI-Tier (Traceability and information system of animals [Herkunftssicherungs- und Informationssystem für Tiere, Bayerisches Staatsministerium für Ernährung, Landwirtschaft, Forsten und Tourismus, Munich, Germany]), such as ear tag number, breed, date of birth, lactation number, and days in milk, were provided for all cows by the farmers before the first visit. Seven farms also allowed access to their official milk yield records.

### 2.3. Recording of Barn Conditions: Construction, Housing Conditions, and Management

Barns were built between 2011 and 2021 as loose housing systems with a concrete walkway and bedded pack resting area. Seven barns were built as new buildings for lactating cows, and one farmer converted an existing barn to a CBP. Seven barns were open front with cross ventilation, and one barn was a closed barn with large windows for ventilation. The most frequently used bedding materials on the eight farms were sawdust (SAD), fine dry sieved wood shavings (WSS), and fine dry wood shavings (WS). In addition, some farmers added small amounts of other materials such as rape straw, spelt hulls, miscanthus, or horse dung. The bedded pack was tilled twice daily (2 ± 0 [median ± MAD]) using a cultivator on five farms and a rototiller on three farms.

A data collection form was used to record barn structures at each visit. It included barn dimensions, lying areas, concrete walkways, and other barn facilities. The barn dimensions were measured with a laser measuring device, which was also used to determine the lying area, concrete walkway, and total barn dimensions separately. The lying area, the concrete walkway, and the outdoor area were evaluated for cleanliness, slip resistance, and condition. A 10 m^2^ section in the centre of the lying area was evaluated for faecal matter. The flooring type and the method, and frequency of cleaning were also evaluated. The cleanliness of the concrete walkway floor was assessed using a 4-point scoring system: 1 = clean floor with single cow pats, 2 = less than 50% of the area is soiled, 3 = more than 50% of the area is soiled, and 4 = the entire area is covered in faeces [10]. A 3-point scoring system was used to evaluate liquid on the floor: 1 = no liquid accumulation, 2 = single puddles of liquid, and 3 = more than 50% of the surface is covered in liquid [10]. Other barn equipment, including fans, scratching brushes, feed-pushing robots, and curtains, were recorded. Two scoring systems were created to evaluate the condition of the lying surface and the concrete walkway: For the lying surface, 3 points = good, 2 points = moderate, and 0–1 points = poor (Supplementary Table S1); for the concrete walkway, 4–5 points = good, 3 points = moderate, and 0–2 points = poor (Supplementary Table S2).

### 2.4. Lameness Scoring and Recording of Claw Lesions

Lameness was scored on a hard surface by the same observer at both visits using a 5-point scale, in which cows with a score of 3 to 5 were considered lame [25]. Foot and claw disorders were recorded during claw trimming. All diagnoses were based on the ICAR Claw Health Atlas and its appendices and were assigned one of three severity scores [26,27,28]. The software KLAUE (version: 3.4.1.3; dsp-Agrosoft GmbH, Ketzin, Germany) was used for digital documentation. This software enables the observer to document claw lesions on the lateral and medial claws and heel horn using a tablet. Cow identification data were imported from the HI-Tier database into KLAUE before the visit.

Claw trimming of the herd was performed by a hoof trimmer on all eight farms. In addition, the farmers also trimmed claws regularly on three farms. Acute claw health problems were treated on most farms by the farmer. The trimming frequency of the herd was on average 2 ± 0 (median ± MAD) times a year. Ongoing claw issues reported by the farmers were DD on one farm and WLD on another. The other six farmers reported no claw health problems. None of the farmers used claw disease prevention procedures such as footbaths.

### 2.5. Evaluation of Compost Quality

The colour and odour of the compost were used to assess its quality. The moisture content of the compost was determined using several semi-quantitative tests as follows (Praxisworkshop Kompostierungsstall, personal communication, 4 July 2022):

In the “ball test” (Praxisworkshop Kompostierungsstall, personal communication, 4 July 2022), compost was pressed into a ball by hand. If the ball fell apart, the moisture content was acceptable, but if the ball remained intact or liquid escaped, the moisture content was considered excessively high. In the “urine test” (Praxisworkshop Kompostierungsstall, personal communication, 4 July 2022), the time required for urine to be absorbed by compost was measured when a cow urinated. Urine absorbed within 30 s indicated an acceptable level of compost moisture, while more than 30 s was too high. In the “boot test” (Praxisworkshop Kompostierungsstall, personal communication, 4 July 2022), compost was compressed by stepping on it for approximately ten seconds. The surface was then evaluated for shine, level of compression, and liquid accumulation. The moisture content was also evaluated by visually assessing the compost surface in the passages to the concrete walkways for shine or dullness. The result was compared with the remaining lying surface. The texture of the compost was evaluated haptically. A 0- to 9-point scoring system was used to grade these semi-quantitative tests; 7–9 points = good, 4–6 points = moderate, and <4 points = poor compost quality (Supplementary Table S3).

For compost sampling, the resting area was divided into nine equally sized plots [29]. In the centre of each plot, a compost sample of approximately 1 kg was taken at a depth of 20 cm. The samples were frozen and later analysed for dry matter [%] at the Institute for Animal Nutrition of the University of Veterinary Medicine Hannover (Germany).

### 2.6. Calculation of the Cow Claw and Farm Claw Scores

To estimate the claw health for individual cows and farms, the cow claw score (CCS) and the farm claw score (FCS) were calculated using Microsoft Excel (Microsoft Corporation, Redmond, WA, USA). This calculation is based on geometric severity scores defined for each claw lesion using three severity grades [30]. The CCS is the sum of all geometric severity scores of all claw and foot disorders for each cow [31,32,33]. The FCS is the median of the CCS of all cows of the respective farm [34].

### 2.7. Statistical Analysis

For statistical analysis, data were collated in Microsoft Excel (Microsoft Corporation, Redmond, WA, USA), and for further analysis, the SAS Enterprise Guide 7.1 (SAS Institute Inc., Cary, NC, USA) was used. The Shapiro–Wilk test was used to test for normality of the distribution of all variables. Mean ± SD were calculated for normally distributed variables and median ± median absolute deviation (MAD) values for variables not normally distributed. The Wilcoxon signed-rank test was used to compare lameness scores in the cold and warm seasons. To evaluate the effect of compost quality, lying surface condition, and concrete walkway condition on lameness, the Kruskall–Wallis test with the Dwass–Steel–Critchlow–Fligner (DSCF) test as post hoc test was conducted. A logistic regression calculated for both seasons was used to estimate the odds ratio of the occurrence of lameness as a function of compost quality, lying surface, and concrete walkway condition. As no control group was available, the values from the PraeRi study [10] were used as comparison values for the prevalence of lameness and the values from an Austrian study [12] for the prevalence of claw disorders. Normally distributed prevalence values were compared with comparison values using a one-sample *t*-test. Differences were considered significant at *p* ≤ 0.05.

## 3. Results

### 3.1. Barn Conditions and Compost Quality

The main reasons to convert to a CBP were animal welfare and health.

The bedded pack resting area was 625 ± 103.5 m^2^ (median ± MAD). The compost-bedded resting area available to each cow was 10.1 ± 1.1 m^2^ (median ± MAD) in the cold season and 9.9 ± 1.2 m^2^ (median ± MAD) in the warm season.

The lying surface condition in the cold and warm seasons was an average of 3/3 ± 0 points (median ± MAD), and the concrete walkway condition was 5/5 ± 0 points (median ± MAD).

The average compost quality was 7.25/9 ± 1.5 points (median ± MAD) in the cold season and 7.75/9 ± 0.75 points (median ± MAD) in the warm season. The analysed dry matter of the compost was 33.6 ± 4.4% (mean ± SD) in the cold season and 39.1 ± 7.4% (mean ± SD) in the warm season.

### 3.2. Lameness Scoring

On an individual cow basis, lameness occurred in 9.4% of the cows (n = 593) in the cold season and in 11.1% (n = 613) in the warm season (*p* ≤ 0.05). At the farm level, the lameness prevalence was 9.5 ± 7.1% (mean ± SD) in the cold season and 10.9 ± 5.8% (mean ± SD) (*p* > 0.05) in the warm season. Farm lameness prevalences were below (*p* ≤ 0.05) the mean comparison value (25.8%) of the PraeRi study [10].

**Figure 1:**
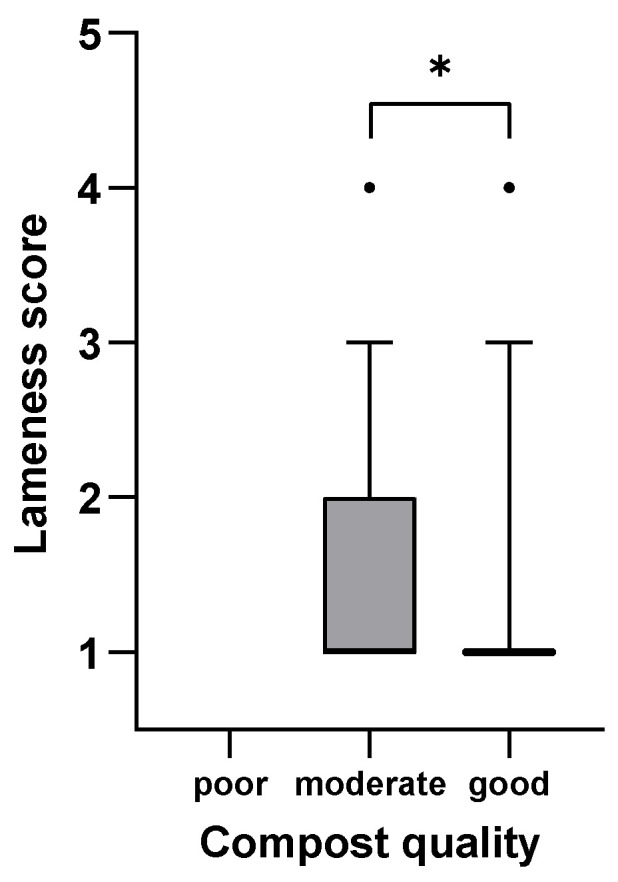
Lameness scores (1–5) of cows in eight CBPs during the cold season (n = 593) under poor (<4 points), moderate (4–6 points), and good (7–9 points; Supplementary Table S3) compost quality conditions expressed as box plots: median; Q 5/95. * Medians of box plots with the same bracket differ (*p* ≤ 0.05).

**Figure 2:**
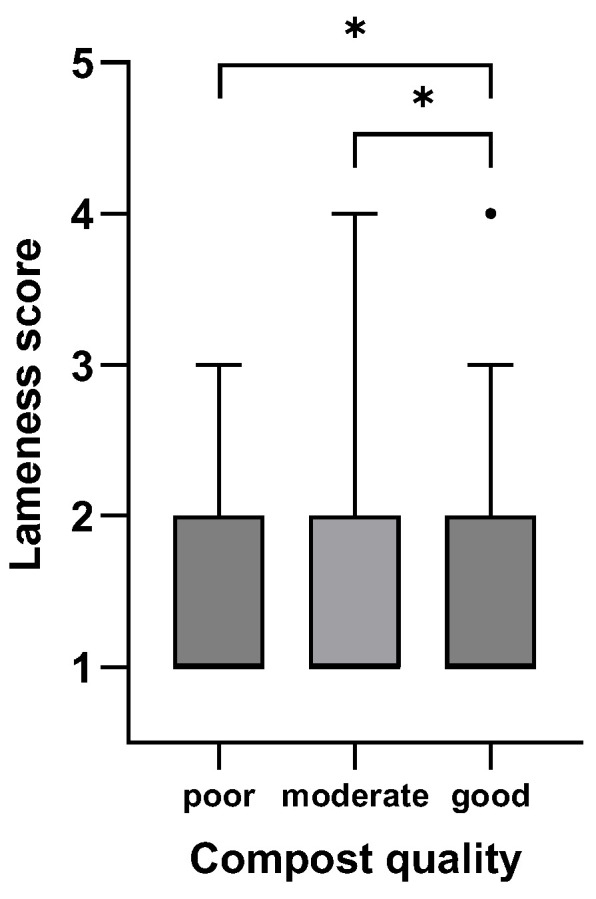
Lameness scores (1–5) of cows in eight CBPs during the warm season (n = 613) under poor (<4 points), moderate (4–6 points), and good (7–9 points; Supplementary Table S3) compost quality conditions expressed as box plots: median; Q 5/95. * Medians of box plots with the same bracket differ (*p* ≤ 0.05).

**Figure 3:**
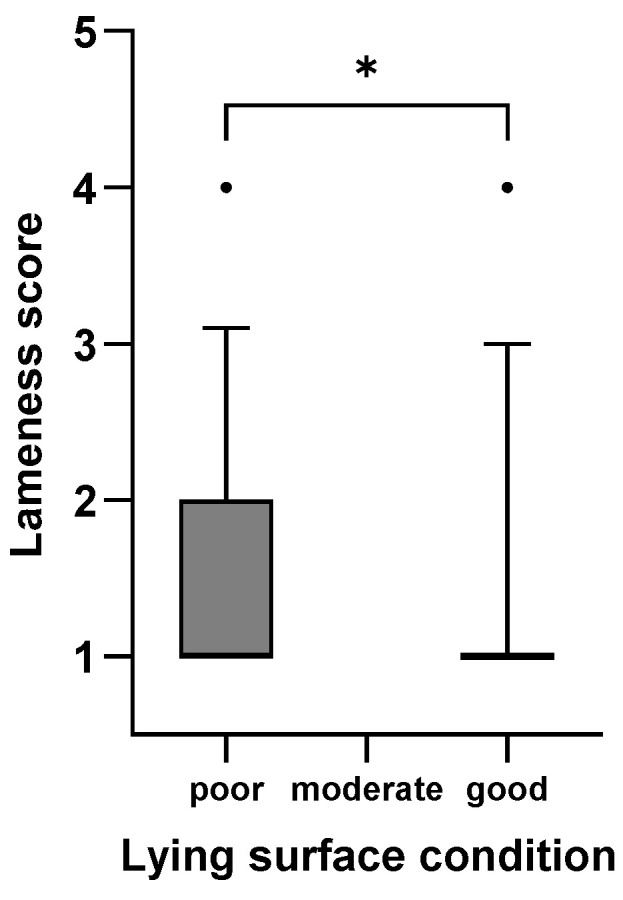
Lameness scores (1–5) of cows in eight CBPs during the cold season (n = 593) under poor (0–1 points), moderate (2 points), and good (3 points; Supplementary Table S1) lying surface conditions expressed as box plots: median; Q 5/95. * Medians of box plots with the same bracket differ (*p* ≤ 0.05).

**Figure 4:**
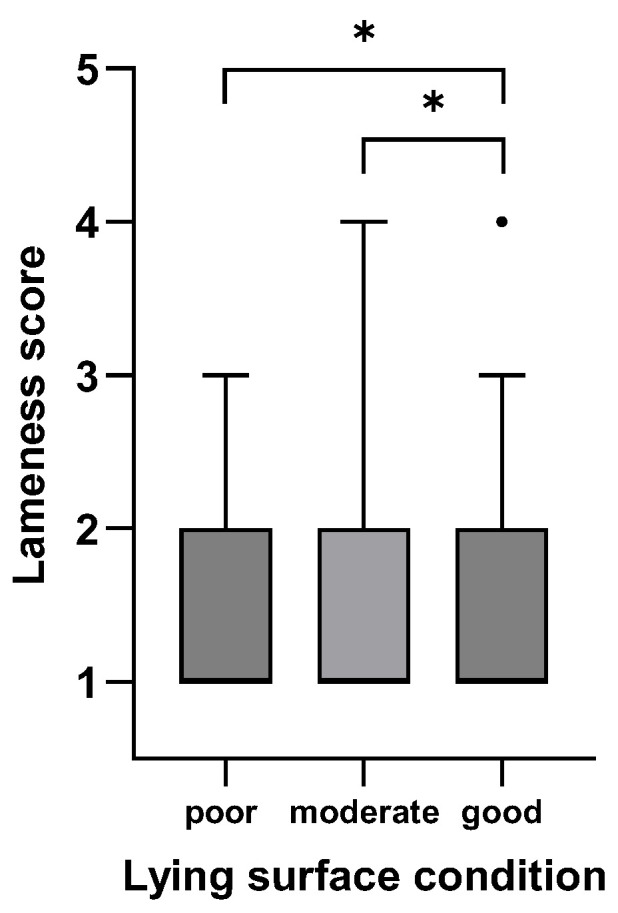
Lameness scores (1–5) of cows in eight CBPs during the and warm season (n = 613) under poor (0–1 points), moderate (2 points), and good (3 points; Supplementary Table S1) lying surface conditions expressed as box plots: median; Q 5/95. * Medians of box plots with the same bracket differ (*p* ≤ 0.05).

**Figure 5:**
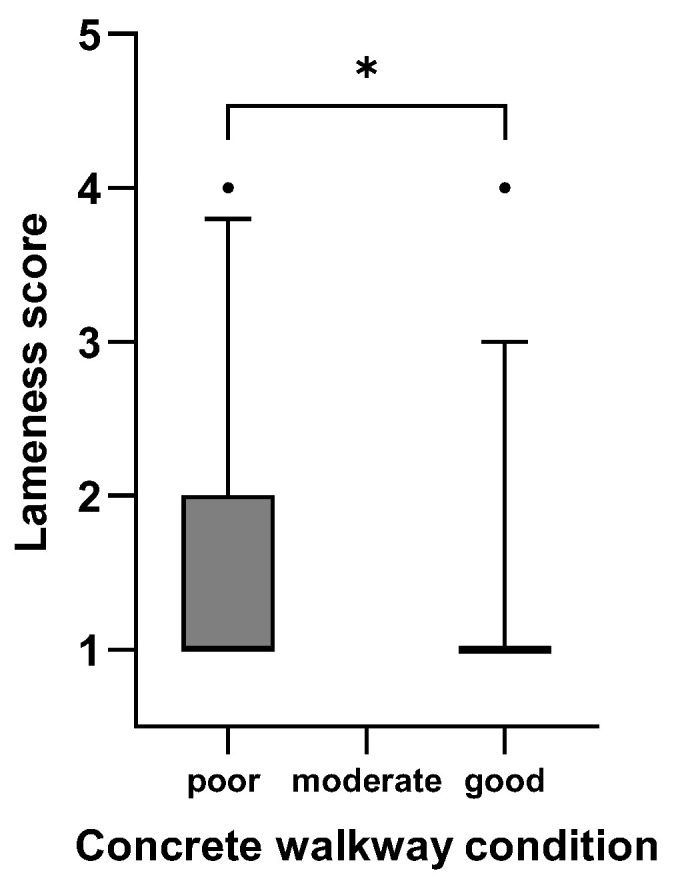
Lameness scores (1–5) of cows in eight CBPs during the cold season (n = 593) under poor (0–2 points), moderate (3 points), and good (4–5 points; Supplementary Table S2) concrete walkway conditions expressed as box plots: median; Q 5/95. * Medians of box plots with the same bracket differ (*p* ≤ 0.05).

**Figure 6:**
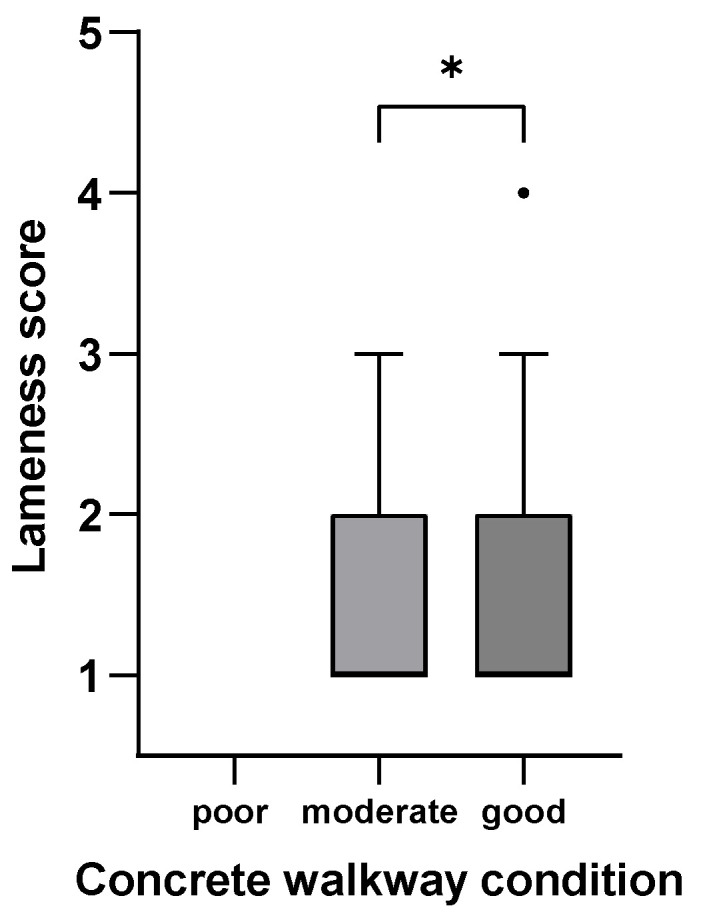
Lameness scores (1–5) of cows in eight CBPs during the warm season (n = 613) under poor (0–2 points), moderate (3 points), and good (4–5 points; Supplementary Table S2) concrete walkway conditions expressed as box plots: median; Q 5/95. * Medians of box plots with the same bracket differ (*p* ≤ 0.05).

Figure 1, Figure 2, Figure 3, Figure 4, Figure 5 and Figure 6: Lameness scores in the cold and warm seasons under various compost quality, lying surface, and concrete walkway conditions.

Only the compost quality in the cold season was associated with the occurrence of lameness. The OR for lameness to occur was 3.7 times higher for cows housed on compost of poor quality than of good quality (Table 1).

### 3.3. Claw Health

#### 3.3.1. Claw Diseases and Claw Shape

Table 2, Table 3 and Table 4 show the prevalences of foot and claw disorders determined during regular claw trimming at cow (n = 640) and farm levels. The scores for each foot and claw disorder recorded during claw trimming are shown.

The most frequent infectious claw disorders were HHE in 42.2% of the cows, followed by DD in 11.3% (Table 2). DD lesions in more than one foot occurred in 25% of the affected cows.

Of the non-infectious foot and claw disorders, white line fissures (WLFs) occurred most often (35.2%), followed by circumscribed sole haemorrhage (SHC) (31.6%) and diffuse sole haemorrhage (SHD) (17.0%) (Table 3).

Asymmetric claws (ACs) were the most common claw shape deviation at 14.4%, followed by corkscrew claws (CCs) at 6.4% (Table 4).

Horizontal horn fissures (HFH), vertical horn fissures (HFV), and interdigital phlegmon (IP) were not seen. Some DD-associated claw horn lesions such as DD-heel horn erosion (DD-HHE), DD-bulb ulcer (DD-BU), DD-horizontal horn fissure (DD-HFH), DD-axial horn fissure (DD-HFA), and DD-toe necrosis (DD-TN) were also not detected.

#### 3.3.2. Cow and Farm Claw Scores

The calculated CCS (n = 640) was 8 ± 8 (median ± MAD), and the FCS (n = 8) was 9 ± 3 (median ± MAD).

## 4. Discussion

Of 30 identified CBP farms, eight met the inclusion criteria and were located mainly in southern Germany, possibly because bedding material is easier to source because of high forest cover [35]. One of the requirements for inclusion in our study was that the CBP had been in place for a minimum of two years, which was similar to a study that compared claw health in CBPs and conventional barns in Austria [24]. Managing a CBP requires experience, and each farmer must develop specific management standards. Two years were chosen to avoid the transition from the old housing system to the CBP, facilitating the determination of the effect of CBP on claw health without including the transition period. The effect of pasture on claw health [36,37] was excluded by selecting farms without access to pasture. A routine claw trimming visit was required to evaluate the claws of the entire herd on one day. Due to the variation in temperature and humidity throughout the year and the seasonal effect on the resting area of the bedded pack [17,38], farms were visited in both the cold and warm seasons.

The average number of cows in the eight herds was 64.5, which was slightly higher than the average of 50.5 in southern Germany [10]. By choosing barns with loose housing, our study population was higher than the regional average including free-stall and usually smaller sized tie-stall barns. The main breed in our study was Fleckvieh (82.2%), which was similar to the PraeRi study, in which 80.4% of the cows in southern Germany were Fleckvieh [10]. The second most common breed was Holstein Friesian (13.4%), in contrast to Brown Swiss in the PraeRi study [10]. This may be due to regional clusters of Brown Swiss cows in Bavaria. The average milk yield was higher (8417.4 kg) in our study than in the PraeRi study (7606.2 kg), but it was similar to the 25% highest yielding PraeRi farms in southern Germany (8456.0 kg).

The CBPs in our study followed designs recommended in previous studies. This included a concrete walkway separated from the bedded pack resting area with waterers on the walkway side of the partition walls to minimise wetting of the pack [15].

The size of the bedded pack resting area per cow is considered one of the most important parameters in CBP design [21]. It affects the amount of bedding material required and thus management, hygiene, and cost. However, its size depends on regional climatic conditions, such as less evaporation in the cold season due to low temperatures and high relative humidity [39]. In 2007, a minimum of 7.4 m^2^ per cow was recommended [15]. Nowadays, a minimum of 8.9 m^2^ to 9.3 m^2^ per cow is recommended [40,41]. However, cows in a close-up pen or maternity area require more space, up to recommended 13.9 m^2^ [40]. Studies in the United States described an average size of 8.6 m^2^ [16], 8.9 m^2^ [42], and 9.0 m^2^ [17]. In Italy, a survey found the bedded pack size to be 6.8 m^2^ per cow (n = 10) [39]. The size of the bedded pack resting area per cow in the present study was 10.1 m^2^ in the cold and 9.9 m^2^ in the warm season. The sizes differed slightly between the cold and warm seasons because of a change in the number of animals, but it may also have practical management reasons. By providing more space per cow, especially in the cold season with low air temperatures and high humidity, less bedding is consumed, facilitating easier management; the risk of overcooling of the bedded pack, which would result in cessation of composting and therefore a wet lying surface, is also reduced [17].

The condition of the lying surface and concrete walkway was good in the cold and warm seasons, showing no seasonal effect on their cleanliness and constructional condition. Assessment of concrete walkways in conventional free-stall cubicle barns revealed poorer conditions, with only 17.7% of the concrete walkways being clean and only 43.3% having good slip resistance [10]. The condition of the concrete walkways in CBPs may be better because, in addition to the walkways, the bedded pack area is also used for exercise [21]. Therefore, walkways may be cleaner because less manure accumulates on them. Management is another possible reason for better walkway conditions in our study.

Our study farms used sawdust, fine dry-sieved wood shavings, and fine dry wood shavings as the main bedding materials, tilled the bedded pack an average of twice daily, and cleaned the pack twice a year, all of which are recommended management practices [15,17,39]. Compost quality was considered good in both seasons but was slightly better in the warm season. This was to be expected because of the poorer weather conditions in the cold season. The lower temperatures cool the bedded pack resting area, leading to poorer biodegradation; the latter affects the pack temperature by inhibiting surface drying. High air humidity prevents the release of water generated during biodegradation into the environment. The resulting humid bedded pack can be ameliorated by adding fresh bedding material more often. Providing more space per cow would be another method of reducing the use of bedding material and demand for biodegradation, keeping the bedded pack dry and biodegradation working [17,38].

Farmers were motivated to transition to CBPs for animal welfare reasons; CBPs provide a high level of cow comfort, thus improving longevity [16]. Farmers commented that they were satisfied with CBPs and the felt main benefits were improved cow comfort [17]. In our study, the most common reason cited by farmers for building a CBP was improved cow comfort and animal health, which was also reflected in good scores for available space per cow, conditions of lying surface and walkways, and compost quality.

Conventional cubicle free-stall barns had lameness prevalences of 22.7% at cow level and 25.8% at farm level, which are higher than the results of the present study (9.4% at cow level and 9.5% at farm level in the cold season and 11.1% at cow level and 10.9% at farm level in the warm season) [10]. The lower prevalence of lameness in CBPs may be attributable to less time standing on hard surfaces and easier lying and rising processes because of unrestricted movement on a bedded pack [16,17,22]. A previous study reported that cows housed in straw-bedded barns had longer lying times than cows in cubicle barns [43]. Increased lying time was also described for cows in CBPs compared with cows housed in free-stall barns with access to pasture [19]. Decreased lying time results in increased standing time, which increases the occurrence of lameness [44]. Although we did not record lying times as part of our study, the increased lying comfort in CBPs may explain the low lameness prevalence.

Concrete is not ideal flooring; its rough surface damages the corium, and it is slippery, leading to abnormal gait patterns [13]. Concrete surfaces are often wet and covered with manure, resulting in claw overgrowth and abnormal weight bearing [45]. Poor concrete hygiene leads to wet claws, which are softer and at higher risk of sole penetration and corium bruising [13]. The concrete walkway hygiene in the present study was good, perhaps contributing to the low prevalence of lameness. We assume that deterioration in walkway management impacts the prevalence of lameness, particularly in the cold season.

In contrast to cubicle barns, cows in CBPs use the bedded pack as an exercise area in addition to the walkways [21]. The bedded pack allows wet claws to dry and provides a resilient surface, into which the claws can sink, fostering normal gait [46].

The prevalence of lameness reported in other studies on CBPs was 4.4% [22], 7.8% [16], and 11.9% [17], which was lower or similar to our results. Lameness prevalences of 18.7% [24], 31.9% [23], and 39.2% [29], which were higher than our results, were reported in three other studies on CBPs. Thus, the lameness prevalence of cows in CBPs can vary widely, similar to conventional dairy cow housing. Differences in compost quality and therefore lying comfort likely play a major role in lameness prevalence.

In the present study, the prevalence of lameness was higher in the warm season compared to the cold season. To the authors’ knowledge, comparable studies on the effect of season on the prevalence of lameness in CBPs are lacking. A previous study reported a reduction in lying time and an increase in standing time between the coolest and hottest study session due to heat stress, with higher lameness scores in the warm season [47]. A study in CBPs indicated, based on climatic variables inside an open CBP, a high stress condition [48].

Thus, a possible reason for the higher lameness prevalence in the warm season is the heat stress during the warm season and thus possibly longer standing times on concrete walkways.

The lameness prevalence in the present study was affected by compost quality and the condition of lying surface and concrete walkways; lower lameness prevalences were seen with higher scores for these variables. Although the prevalence of lameness differed statistically in both seasons, only the difference in the cold season can be considered meaningful (Figure 1, Figure 2, Figure 3, Figure 4, Figure 5 and Figure 6). Good-quality compost was drier, softer, and finer than poor-quality compost. Lying time was shown to be reduced on muddy, wet, and manure-contaminated surfaces [49,50], and cows prefer to lie down and lie longer on soft [51] and dry surfaces [52]. Less standing time can reduce the occurrence of lameness [44]. Furthermore, a dry lying surface is essential for claws to dry; dry claws are harder than wet claws, leading to a lower risk of sole penetration and corium bruising [13]. Compost quality was the only significantly associated factor with an odds ratio of 3.7, highlighting the importance of this parameter.

Our cow-level prevalence of HHE, an infectious claw and foot disorder, was 42.2%, which was lower than that of the comparison study (48.0%) [12] and a Swiss study (64.2%) [51], both of which used cows kept in conventional housing systems. It has been shown that the prevalence of HHE is lower in cows housed in clean conditions than in cows with high exposure to manure and urine [53]. The prevalence of HHE was 26.9% of cows housed in CBPs in Austria, which was lower than our result [24]. However, both studies suggest the lower prevalence of HHE is due, at least in part, to the soft, dry, clean surface of the bedded pack [24].

The second most common infectious claw and foot disorder was DD, which has a high prevalence worldwide. The prevalence of DD in the present study was 11.3% at cow level, 10.7% at farm level (n = 8), and 14.3% for the affected herds (n = 6). In comparison, the prevalence of DD in conventional housing systems was 0 to 56.2% at herd level and 24.1% at cow level in Denmark [53], 0 to 87.5% at herd level and 20.7% at cow level in Switzerland [54], and 18.6% at herd level and 33.2% for all affected herds in Austria [12]. The values in the latter study were used as a comparison. Thus, conventional free-stall housing systems favoured a markedly higher prevalence of DD than CBPs. Risk factors for DD include contact of claws with manure [55], making frequent manure scraping an important method for reducing this disease [56,57]. Reduced time on manure-covered walkways [19] and ensuring optimum walkway conditions would likely lower the prevalence of DD. Cows housed in a straw-bedded yard had a lower DD prevalence than those housed on other floor types [58], which supported the results of another study that found low survivability of *Treponema* spp. in straw [59]. Similar studies have not been reported for cows housed in CBPs. In addition, the bedding material in CBPs may clean the interdigital space mechanically, allowing the area to dry.

Interdigital phlegmon did not occur in our study. Other studies reported prevalences of 0.8% [12] and 0.2% [54] in cows housed in conventional housing systems. Predisposing factors for IP include damp soil and injuries to the interdigital skin [60], which allow entry of *Fusobacterium necrophorum*, the main pathogen of the disease [61]. The good housing conditions in the CBPs may have prevented skin injury and subsequent IP in our study.

The most common non-infectious foot and claw disorders were WLF (37.1%), DS (8.0%), ulcers (4.7%), and IH (2.2%). In comparison, the comparison study of dairy cows in conventional housing systems in Austria had higher values for WLD (56.8%), DS (18.8%), ulcers (13.6%), and IH (5%) [12]. Sole haemorrhage was also common in the present study with 31.6% of the cows having SHC and 19.2% SHD, which was higher than the comparison study (29.6%) involving conventional housing systems [12]. The higher prevalence of sole haemorrhage may have been attributable to nutritional problems [62] or better observation and diagnosis by the observers. The sample size of the farms in our own study was much smaller than in the studies used for comparison [12,54]. Therefore, only a limited statement can be made here.

One study concluded that the lower prevalence of claw disorders in CBPs compared with conventional free-stall barns was due to the soft, dry, clean bedded pack and concrete being limited to the walkways [24]. Studies have reported that the claws incur lower pressure loads on softer surfaces than concrete flooring [61,62]. This reduces the risk of mechanically induced claw lesions [63,64]. In addition, one study found that the force and pressure distribution on the claws of cows walking on hard surfaces was a potential risk to claw integrity [65]. Claw contact with manure and urine softens the skin and the claw horn making it more vulnerable to mechanical insults [66]. More severe claw lesions were seen in softer claws and thus providing conditions that maximise dry claws was recommended to reduce the likelihood of claw injuries [67].

A CCS and FCS of <35 have been defined as threshold values for good claw health [31,68]; a CCS of <15 was considered excellent claw health, 16–30 as mildly, 31–100 as moderately, and >100 as severely compromised claw health [24]. The CCS of the present study was 8, which is in the excellent range [24]. A recent study of 508 conventional free-stall and tie-stall dairy herds in Austria reported a median CCS of 24 for Fleckvieh and 22.7 for Holstein Friesian, notably higher values than those of the present study [12]. Lower CCS values were reported for cows housed in CBPs compared with cows in free-stall cubicle barns [24]. Our FCS was 9, which is markedly lower than the threshold value of <35 defined in previous studies [31,68] and the threshold value of <20, reported as the median of 508 dairy farms [12]. Another study showed lower FCS values for CBP herds than those in free-stall cubicle barns [24]. Our CCS and FCS values reflected a low prevalence of lameness and foot and claw disorders. Both values allow the user to compare claw health across studies in an objective standardised manner.

## 5. Conclusions

Compared with conventional housing systems, the prevalence of lameness and claw disorders was lower in CBPs, with a low CCS and FCS, revealing that this alternative housing system may reduce lameness and promote claw health. It is important to note that the awareness and motivation of the farmers to improve animal welfare and health by choosing CPBs and optimising barn and cow management were crucial to achieving these results.

## Figures and Tables

**Table 1:** Results of the logistic regression to estimate the odds ratio of the occurrence of lameness as a function of compost quality, lying surface, and concrete walkway condition.

	Group Comparison	Cold Season			Warm Season		
		OR	*p*-Values	95% Wald Confidence Interval	OR	*p*-Values	95% Wald Confidence Interval
Compost quality	moderate vs. good	3.7	0.0001	1.9–7.3	1.8	0.0861	0.9–3.6
	poor vs. good				0.9	0.9037	0.4–2.5
Lying surface	moderate vs. good				1.8	0.0861	0.9–3.6
	poor vs. good	0.8	0.6070	0.3–1.9	0.9	0.9037	0.4–2.5
Concrete walkway	moderate vs. good				1.8	0.0861	0.9–3.6
	poor vs. good	1.2	0.5821	0.5–3.1			

OR = odds ratio.

**Table 2:** Prevalence of infectious foot and claw disorders in cows (n = 640) from eight CBPs recorded during regular claw trimming at cow and farm levels. Farm-level values were compared to comparison values of an Austrian study [12].

Claw Disorder	Total [%]		Severity Score [%]			Comparison Values [%]	*p*-Values
	Cow Level	Farm Level	Slight	Moderate	Severe		
HHE	42.2	38.5 ± 26.7 ^#^	94.5	5.1	0.4	48.0	0.3465
DD ^a^	11.3	10.7 ± 12.1 ^#^	n.a.	n.a	n.a	18.6	0.1087
DD ^b^	11.3	14.3 ± 12.0 ^#^	n.a.	n.a.	n.a.	33.2	0.0119
ID	1.9	0 ± 0 *	n.a	n.a	n.a	n.a.	n.a

HHE = heel horn erosion, DD = digital dermatitis, ID = interdigital dermatitis, n.a. = not applicable. * Median ± MAD, ^#^ mean ± SD, ^a^ all herds (n = 8), ^b^ DD-affected herds (n = 6).

**Table 3:** Prevalence of non-infectious foot and claw disorders in cows (n = 640) from eight CBPs recorded during regular claw trimming at cow and farm levels. Farm-level values were compared to comparison values of an Austrian study [12].

Claw Disorder	Total [%]		Severity Score [%]			Comparison Values [%]	*p*-Values
	Cow Level	Farm Level	Slight	Moderate	Severe		
WLF	35.2	37.1 ± 19.1 ^#^	90.3	9.4	0.3	56.8	0.0266
SHC	31.6	31.6 ± 10.7 ^#^	93.6	6.4	0.0	n.a.	n.a.
SHD	17.0	19.2 ± 16.9 ^#^	86.5	12.9	0.6	n.a.	n.a.
DS	7.3	8.0 ± 7.0 ^#^	59.2	34.7	6.1	18.8	0.0033^2^
Ulcers (SU, BU, TU)	4.5	4.7 ± 4.7 ^#^	n.a.	n.a.	n.a.	13.6	0.001
SU	3.4	3.4 ± 3.5 ^#^	73.9	21.7	4.4	n.a.	n.a.
IH	2.5	2.2 ± 1.9 ^#^	84.7	5.3	0.0	5.0	0.012
HFA	1.9	0.9 ± 0.9 *	82.4	17.6	0.0	n.a.	n.a.
WLA	1.4	0 ± 0 *	70.0	30.0	0.0	n.a.	n.a.
SW	1.1	0 ± 0 *	100.0	0.0	0.0	n.a.	n.a.
BU	0.8	0 ± 0 *	80.0	0.0	20.0	n.a.	n.a.
TU	0.2	0 ± 0 *	0.0	100.0	0.0	n.a.	n.a.
TN	0.2	0 ± 0 *	n.a.	n.a.	n.a.	n.a.	n.a.

WLF = white line fissure, SHC = circumscribed sole haemorrhage, SHD = diffuse sole haemorrhage, DS = double sole, SU = sole ulcer, IH = interdigital hyperplasia, HFA = axial horn fissure, WLA = white line abscess, SW = swelling of the coronet and/or bulb, BU = bulb ulcer, TU = toe ulcer, TN = toe necrosis, n.a. = not applicable. * Median ± MAD, ^#^ mean ± SD.

**Table 4:** Prevalences of claw shape deviations in cows (n = 640) from eight CBPs recorded during regular claw trimming at cow and farm levels.

Claw Disorder	Total [%]	
	Cow Level	Farm Level
AC	14.4	16.8 ± 9.3 ^#^
CC	6.4	1.3 ± 1.3 *
SC	1.6	0.8 ± 0.8 *
CD	1.1	0.4 ± 0.4 *
TS	0.8	0 ± 0 *

AC = asymmetric claws, CC = corkscrew claws, SC = scissor claws, CD = concave dorsal wall, TS = thin sole. * Median ± MAD, ^#^ mean ± SD.

## Data Availability

The original contributions presented in this study are included in the article/Supplementary Material. Further inquiries can be directed to the corresponding author.

## Supplementary Materials

The following supporting information can be downloaded at: https://www.mdpi.com/article/10.3390/ani15091347/s1, Table S1: Scoring system to evaluate the lying surface condition; Table S2: Scoring system to evaluate the concrete walkway condition; Table S3: Scoring system to evaluate the compost quality.

## Published Materials

This article is a revised and expanded version of a conference paper entitled “Lameness and claw health of dairy cows in German compost bedded pack barns”, which was presented at the World Buiatrics Congress, Cancun, Mexico, 20–24 May 2024 [69] and a conference paper entitled “Claw health and lameness in German compost bedded pack barns”, which was presented at the 22nd International Symposium and 14th International Conference on Lameness in Ruminants, Venice, Italy, 16–20 September 2024 [70].
