# Supplementary material for: The Effect of Compost-Bedded Pack Barns on Claw Health and Lameness in Dairy Herds in Southern Germany
Source: Animals (Basel). 2025 May 7;15(9):1347. doi: 10.3390/ani15091347 (PMC12071046; doi:10.3390/ani15091347)
Supplement: Supplementary file 1 [file animals-15-01347-s001.zip › animals-3575994-supplementary.pdf]

Supplementary Table 1: Scoring system to evaluate the lying surface condition

Parameter	Result	Points	Result	Points	Result	Points
**Lying surface cleanliness**	clean	1	soiled	0		
**Cow pats/10 m^2**	0-2	1	>2	0		
**Lying surface condition**	good	1	moderate	0.5	poor	0

Supplementary Table 2: Scoring system to evaluate the concrete walkway condition

Parameter	Result	Points	Result	Points	Result	Points
**Concrete walkway cleanliness**	clean	1	soiled	0		
**Concrete walkway slip resistance**	good	1	poor	0		
**Concrete walkway condition**	good	1	moderate	0.5	poor	0
**Concrete walkway amount of manure**	clean, single cow pats	1	<50% of the area covered	0.5	>50% of the area covered	0
**Concrete walkway amount of liquid**	no puddles/a few puddles	1	<50% covered with liquid	0.5	>50% covered with liquid	0

Supplementary Table 3: Scoring system to evaluate the compost quality

Parameter	Result	Points	Result	Points	Result	Points
**Compost surface**	dull	2	matt to glossy	1	glossy	0
**Ball test**	ball disintegrates	1	ball stays intact	0		
**Boot test**	light compaction with matt surface	1	compaction with glossy surface	0.5	compaction with liquid accumulation	0
**Urine test**	drains within 30sec	1	drains in >30sec	0		
**Odor**	peaty, woody, earthly, forest-floor like	2	ammonia-like or manure-like odor	0		
**Hand texture**	fine crumbly	1	firm lumpy	0		
**Passageways**	same as the remainder of the surface	1	compacted	0.5	compacted and wet	0
